# Associations of T-Cell Receptor Repertoire Diversity with L-Asparaginase Allergy in Childhood Acute Lymphoblastic Leukemia

**DOI:** 10.3390/cancers15061829

**Published:** 2023-03-17

**Authors:** Shawn H. R. Lee, Zhenhua Li, Evelyn H. Z. Lim, Winnie H. N. Chin, Nan Jiang, Kean Hui Chiew, Zhiwei Chen, Bernice L. Z. Oh, Ah Moy Tan, Hany Ariffin, Jun J. Yang, Allen E. J. Yeoh

**Affiliations:** 1 Department of Pharmaceutical Sciences, St Jude Children’s Research Hospital, Memphis, TN 38105, USA; 2 Department of Pediatrics, Yong Loo Lin School of Medicine, National University of Singapore, 1E Lower Kent Ridge Road, Tower Block Level 12, Singapore 119228, Singapore; 3 Khoo Teck Puat-National University Children’s Medical Institute, National University Health System, Singapore 119074, Singapore; 4 Department of Pediatrics, KK Women and Children’s Hospital, Singapore 229899, Singapore; 5 Department of Pediatrics, University of Malaya Medical Centre, Kuala Lumpur 59100, Malaysia

**Keywords:** T-cell receptor repertoire, L-asparaginase, allergy, hypersensitivity, childhood acute lymphoblastic leukemia

## Abstract

**Simple Summary:**

Asparaginase allergy is the most common side effect of childhood acute lymphoblastic leukemia (ALL) therapy and may affect survival outcomes, but the basis of T-cell responses—especially the T-cell receptor (TCR) repertoire—is unknown. The aim of this study was to characterize the associations of TCR repertoire diversity with the risk of allergy. We found that a TCR repertoire that was more varied and changed more during therapy was associated with an increased risk of clinical allergy and decreased asparaginase activity later in therapy. A higher variability of the repertoire between timepoints was predictive of the occurrence of allergy. After an allergy had occurred, the TCR repertoire became altered and more similar. Understanding the immunological basis of this common toxicity in childhood ALL helps in the development of more effective methods to predict or mediate this event.

**Abstract:**

Asparaginase is a critical component of therapy for childhood acute lymphoblastic leukemia (ALL), but it is commonly associated with allergy, which results in morbidity and poorer outcomes. The underlying basis of this allergy is undoubtedly immune-mediated, but the exact components of T-cell immunity have yet to be characterized. We performed longitudinal TCR sequencing of 180 bone marrow samples from 67 children with B-ALL treated as part of the Ma-Spore-ALL-2010 trial, and we evaluated the associations of TCR profile with asparaginase hypersensitivity, with functional validation of asparaginase activity in a separate cohort of 113 children. We found that a more diverse and dynamically changing TCR repertoire was associated with increased risk of clinical hypersensitivity and decreased L-asp activity. Allergic patients had a higher proportion of infrequent clonotypes, as well as a significantly lower degree of shared clonotypes amongst the cohort. Allergic patients also had significantly higher longitudinal variability of clonotypes across timepoints, where a higher dissimilarity between diagnosis and week 5 represented an 8.1-fold increased risk of an allergic event. After an allergy had occurred, there was shaping and convergence of the TCR repertoire towards a common antigen. Understanding the immunological basis of T-cell responses in allergy lays the groundwork for developing predictive biomarkers or strategies to mediate this common toxicity in childhood ALL.

## 1. Introduction

Acute lymphoblastic leukemia (ALL) is the most common childhood malignancy. Asparaginase is an indispensable drug in the therapy of this cancer and is used in virtually all contemporary ALL protocols across the globe [1]. Treatment with asparaginase is associated with a plethora of adverse effects, amongst which the occurrence of hypersensitivity is the most common, affecting as many as 70% of children in some treatment protocols [2,3]. Hypersensitivity, whether overt or silent, is associated with dose limitations that, in turn, may result in poorer outcomes—especially if there is no suitable replacement [4,5,6]. There are three main formulations: (1) *E. coli*-derived native L-asparaginase (L-asp), which is used in South East Asia, Central America, and most developing countries because of its lower cost [7,8,9,10]; (2) pegaspargase (PEGasp), where L-asp is conjugated with polyethylene glycol (PEG), which allows less frequent dosing and is generally the first-line treatment in the USA and Europe; and (3) Erwinia asparaginase (Erwinase), isolated from Erwinia chrysanthemi, which is used in patients who have developed hypersensitivity to the other E. coli asparaginase preparations. Although rates of allergy are generally lower with PEGasp, hypersensitivity still remains a significant morbidity in these patients [11]. More importantly, most childhood ALL protocols around the world continue to utilize L-asp, especially in lower–middle-income countries (LMICs). Therefore, further strategies to mediate the occurrence of dose-limiting hypersensitivity to L-asp are needed [12]. Moreover, hypersensitivity is often unpredictable. For this reason, the lack of effective predictive biomarkers for hypersensitivity remains a barrier to improving cure rates and quality of life in these patients.

The basis of hypersensitivity is undoubtedly immune-mediated [4,11,13,14]. PEGylation diminishes the immunogenicity of L-asp, and the antibodies in children receiving PEGasp appear to be mostly directed towards PEG rather than towards asparaginase itself [11]. In both formulations, although antibodies have been found to be predictive of hypersensitivity, the sensitivity and specificity of these antibodies are only modest [4,15]. Moreover, a substantial proportion of events occur in those with no anti-asparaginase or anti-PEG antibodies, pointing to immune mechanisms beyond antibody-mediated responses [4,11]. Previous genomic association studies by us and others have found that strong risk factors for asparaginase allergy include variants within genes regulating T-cell immune response, and that this is agnostic of asparaginase preparation [13,16]. Inhibition of the pathways associated with these variants results in attenuation of hypersensitivity in vivo [12]. Furthermore, multiple studies in diverse ethnic populations have found associations with asparaginase hypersensitivity in both class I and class II HLA alleles [17,18,19]. These findings indicate the clear role of T cells in the pathophysiology of asparaginase allergy; however, the exact aspects of T-cell immunity underpinning this association remain unknown.

T-cell recognition of antigens is arguably one of the most important facets for establishing an immune response, of which a crucial component is the repertoire and diversity of T-cell receptors (TCRs) [20,21]. Although there is growing evidence to suggest the role of TCR repertoires in drug allergy, autoimmunity, and various immune-mediated conditions [22,23,24,25,26], the role of the TCR repertoire in L-asp allergy has yet to be characterized.

A deeper understanding of the aspects of T-cell immunity involved in L-asparaginase allergy provides the basis for therapeutic interventions or strategies to modulate this condition. Therefore, we sought to evaluate the role of the TCR repertoire and its diversity in L-asp hypersensitivity in children undergoing treatment for ALL. To this end, we longitudinally profiled the TCR repertoires in children with de novo ALL treated as part of a frontline ALL trial, with functional validation in a separate cohort, and evaluated these TCR characteristics to determine their associations with asparaginase hypersensitivity.

## 2. Materials and Methods

### 2.1. Patient Cohort

**Figure 1:**
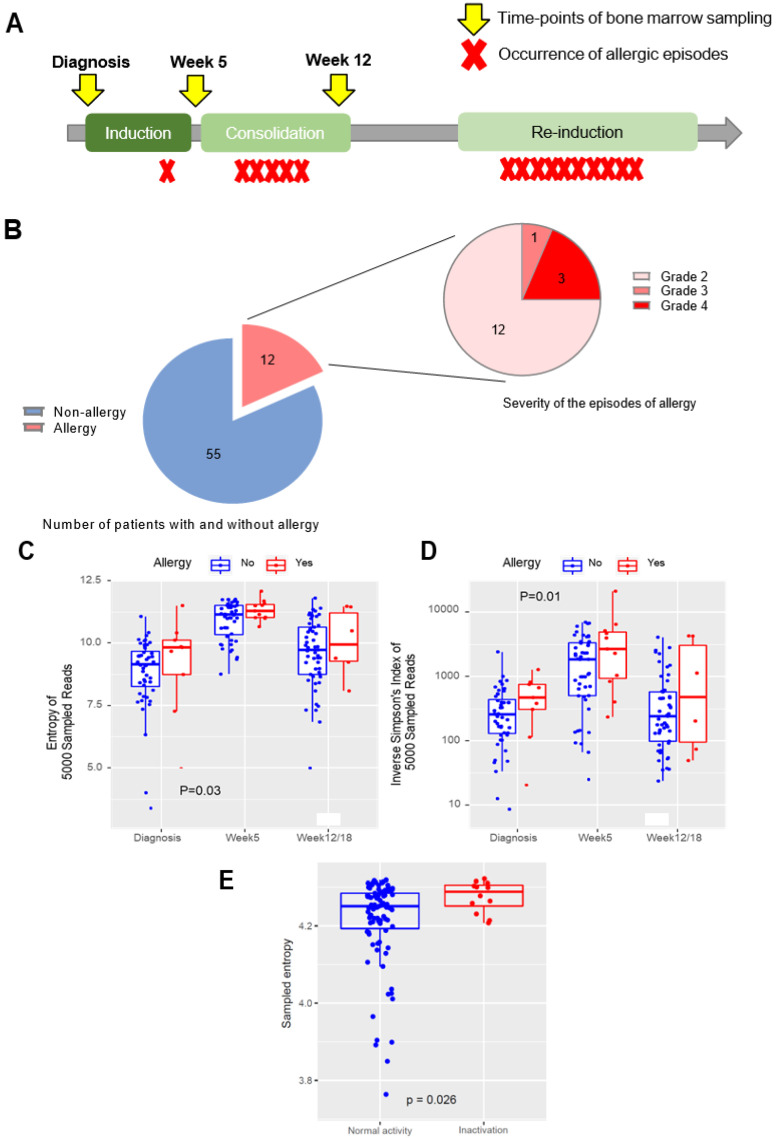
L-asparaginase allergy in childhood ALL, and the association of TCR-B diversity with occurrence of subsequent allergy: (**A**) Overall study schema. The phases of chemotherapy in the Ma-Spore 2010 protocol are shown, with timepoints of bone marrow sampling indicated by yellow arrows and occurrences of allergic episodes indicated by red crosses. (**B**) Number of patients with allergy in the cohort, and the distribution of allergy grades amongst the allergy episodes. The left pie chart depicts the proportion of patients with allergy (in red), and the right pie chart depicts the distribution of severity of 16 episodes of allergy. (**C**) Shannon’s entropy in non-allergy vs. allergy groups throughout all timepoints. Shannon’s entropy is plotted for each patient in the scatterplot at each timepoint and compared between pre-allergic and non-allergic patients; *p*-values determined by the Mann–Whitney test. (**D**) Inverse Simpson’s index in non-allergy vs. allergy groups throughout all timepoints. The inverse Simpson’s index is plotted for each patient in the scatterplot at each timepoint and compared between pre-allergic and non-allergic patients. Both Shannon’s entropy and the inverse Simpson’s index are higher in pre-allergic patients at all timepoints, indicating a higher TCR repertoire diversity in this group of patients who would later go on to develop allergy. *p*-Values determined by the Mann–Whitney test. (**E**) Shannon’s entropy in normal L-asp activity vs. L-asp inactivation in the functional validation cohort. Shannon’s entropy is plotted for each patient in the scatterplot and compared between patients with normal L-asp activity (blue) and patients with L-asp inactivation (red). L-asp inactivation is defined as an activity level of <100 U/L. *p*-Values determined by the Mann–Whitney test. For (B), (C), and (D), patients without allergy are indicated in blue, and patients who would go on to develop allergy are indicated in red. The median of each group is indicated by a bold horizontal line in the boxplot.

The main study cohort comprised 67 children with newly diagnosed B-ALL treated as part of the Malaysia–Singapore 2010 (Ma-Spore 2010, Clinicaltrials.gov NCT02894645) study [27], of which 12 patients had L-asp hypersensitivity, with a total of 16 episodes. Bone marrow aspiration samples were obtained at diagnosis, the end of induction (week 5), and the end of consolidation (week 12) (Figure 1A). These samples were banked in the NUS-Viva CenTRAL Leukemia Tissue Bank. Patients and samples were included in this study on the basis of the availability of banked bone marrow samples with corresponding annotated clinical data. The functional validation cohort consisted of 113 patients from the Malaysia–Singapore 2020 (Ma-Spore 2020) protocol. This study was approved by the respective institutional review boards, and written informed consent was obtained from parents, guardians, and/or patients, as appropriate.

### 2.2. L-Asparaginase Therapy and Measurement of L-asp Activity

The detailed treatment regimen of the Ma-Spore 2010 protocol has been described previously [27]. Briefly, for induction therapy (protocol 1A), L-asparaginase was administered intramuscularly at a dose of 7500 u/m2 twice a week, for a total of 8 doses (4 weeks). In consolidation (protocol 1b#1), one dose of L-asp was given at a dose of 7500 u/m2/dose on day 1. Based on end-of-induction and end-of-consolidation minimal residual disease (MRD), patients were stratified into 3 risk arms: standard risk (SR), intermediate risk (IR), and high risk (HR). Patients on the SR arm received 2 blocks of reintensification, each consisting of 5 doses of L-asp at 10,000 u/m2/dose on days 3, 6, 9, 12, and 15 (10 doses total). Patients in the IR and HR arms both received 3 blocks of reintensification (or 15 doses of L-asp total). Patients who exhibited clinical allergy were either rechallenged at the physician’s discretion or given substitution with Erwinia asparaginase (Erwinase). Those who failed rechallenge were switched to Erwinase. If clinical allergy was confirmed for both forms, asparaginase was discontinued.

L-asparaginase inactivation is a functional marker of both overt and subclinical (or silent) hypersensitivity [28]. We measured L-asp activity from 508 serum or plasma samples from 113 Ma-Spore 2020 patients on days 15 and 29 during induction (phase 1a), and on days 8 and 15 during reintensification (phases III and V, respectively), using a previously published method [11]. L-asp inactivation was defined as having a low L-asp activity level of <100 U/L.

### 2.3. Phenotyping of Clinical Allergy to Asparaginase

The allergic reactions to asparaginase were characterized by local symptoms (i.e., pain, swelling, erythema) and/or systemic manifestations (fever > 38.0 degrees Celsius, urticaria, or edema) and graded using the National Cancer Institute Common Toxicity Criteria (NCICTC version 3.0). Only patients who developed grade 2 or higher reactions were included in this study. Drug substitution was performed at the attending physician’s discretion.

### 2.4. T-Cell Receptor Sequencing and Generation of TCR Repertoires

Bone marrow mononuclear cells (BMMCs) from the bone marrow aspirate were obtained by Ficoll-Paque density gradient centrifugation. DNA was isolated using the TRIzol (Thermo Fisher Scientific, Waltham, MA, USA) extraction method and quantified using a spectrophotometer (NanoDrop 2000, Thermo Scientific). DNA-based targeted sequencing of the TCR beta (TRB) and TCR gamma (TRG) repertoires was performed using the LymphoTrack TRB and TRG Assay Panels (Invivoscribe, San Diego, CA, USA). An input of 1 μg of genomic DNA was used to generate an amplicon library based on the one-step PCR preparation method recommended by the manufacturer. Generated amplicons were purified using AMPure beads (Agencourt AMPure XP; Beckman Coulter). The yield of the purified products was checked by using the DNA High-Sensitivity chip on the Agilent Bioanalyzer (Agilent, Waldbronn, Germany). Amplicon quantification was then performed with KAPA library quantitation kits (KAPA Biosystems). Then, 4 nM of the purified library was sequenced on a NextSeq 500 system (Illumina, San Diego, CA, USA) using the NextSeq 500/550 Mid Output Kit v2.5 (300 Cycles) (20024905, Illumina) at the National University of Singapore, with paired-end read lengths of 2 × 151 bp. For patients from the Ma-Spore 2020 cohort, TCR repertoires were derived from RNA sequencing performed on diagnostic bone marrow samples, as described previously [29].

### 2.5. Analysis of T-Cell Receptor Repertoire Diversity and Statistical Analysis

Sequencing reads were mapped to germline TCR V, D, and J gene segments and clustered into clonotypes using MIXCR [30]. TCR clonotypes were defined as each unique complementarity-determining region 3 (CDR3) amino acid sequence. Disease clonotypes (defined as >5% frequency and with at least 500 reads in diagnostic samples) were removed from the diagnostic samples and subsequent follow-up samples. Only clonotypes that were productive and with more than 5 reads were kept for downstream analysis. Samples with less than 5000 reads were excluded. After filtering and quality control, the absolute number of reads and the number of productive clonotypes were generated for each sample. TCR repertoire diversity and TCR clonality scores for each sample were calculated at each timepoint. To calculate the TCR diversity, the inverse Simpson index of diversity and the Shannon–Wiener entropy index were used [31,32,33]. As these indices are affected by sequencing depth, 5000 reads were sampled 200 times, and the average values were used. At all timepoints, these indices were assessed against the occurrence of hypersensitivity. The similarity of TCR repertoires of the same patient between two timepoints was measured by the Bhattacharyya coefficient, defined as $S=\sum_{i=1}^{k}\sqrt{P_{ia}P_{ib}}$, where k is the number of shared clonotypes between timepoint a and timepoint b, and $P_{ij}$ is the proportion of the *i*th shared clonotype at timepoint j. The distance between two CDR sequences was defined by the restricted Damerau–Levenshtein distance—the number of substitutions, insertions, transpositions, and/or deletions necessary to transform one sequence into another—and was normalized by the length of the longer amino acid sequence. Shared clonotypes were defined as clonotypes that appeared in more than 10% of the patients. Public clonotypes were defined as sequences that are shared in the general population and reported in VDJdb [34] (retrieved on 20 February 2021).

For the validation cohort (Ma-Spore 2020), TRB reads were extracted from the raw RNA-Seq reads and clustered using MIXCR [30]. Minor changes to the workflow were adopted compared to the targeted TCR sequencing above, due to the lower coverage of TRB clonotypes from RNA sequencing. Specifically, disease clonotypes with more than 5% and 10 reads were excluded. Sampled entropy was calculated by resampling 20 reads from the repertoire 5000 times.

Comparisons of continuous variables (TCR diversity, similarity, distance, etc.) between groups were made with the two-sided Mann–Whitney test. Fisher’s exact test was used to determine the differences in the frequencies of categorical variables (e.g., gender, risk group, occurrence of allergy). Statistical analyses were performed using R4.0.3 (http://www.r-project.org). *p*-Values were two-sided unless otherwise indicated, and *p*-values < 0.05 were considered to be statistically significant.

## 3. Results

### 3.1. Clinical Characteristics of L-asp Hypersensitivity

Among the 67 patients (with a total of N = 180 samples across three timepoints) on whom we performed TCR sequencing, 12 patients (17.9%) had L-asp hypersensitivity, representing 16 episodes in total, with 4 repeated episodes occurring in 3 patients during subsequent rechallenges (Table 1 and Figure 1B). Three episodes were grade 4 (19%), one episode was grade 3 (6%), and the remainder were grade 2 (75%). Nine patients were switched to Erwinase, and three patients were had L-asp discontinued at the attending physician’s discretion. Six episodes (37%) were “early” allergies occurring in induction or consolidation therapy, and the remaining ten episodes (63%) occurred during reintensification therapy (“late” allergies). Younger patients tended to have allergy more frequently compared to older patients (*p* = 0.001); otherwise, there were no differences in clinical characteristics between allergic and non-allergic patients in our cohort (Supplementary Table S1). There was a higher proportion of allergy in the SR arm, consistent with other study groups where allergy was also more frequent in their reduced-intensity arms [4,35].

### 3.2. Higher TCR-Beta Diversity at All Earlier Timepoints Is Associated with the Occurrence of Future Allergy and L-Asparaginase Inactivation

We first hypothesized that increased TCR diversity early in therapy would be associated with the occurrence of subsequent allergies because of a more reactive immunological milieu. Therefore, we first evaluated the TCR repertoire diversity in the earlier periods of treatment and the relationship with future (i.e., late-occurring) allergies. Here, we found that the occurrence of allergy was significantly correlated with higher TCR-beta (TRB) diversity at all earlier timepoints as defined by Shannon’s entropy (*p* = 0.03; Figure 1C) or the inverse Simpson’s index (*p* = 0.01; Figure 1D), with the strongest associations noted for the diagnosis timepoint compared to week 5 or week 12. There were no differences in TRB reads at any timepoint (*p* = 0.63) (Supplementary Figure S1A); thus, the difference in diversity was not attributable to increased sampling. We also concurrently tested TCR gamma (TRG) for each sample along with TRB, but we found that there were no differences in diversity for TRG across allergic phenotypes (Supplementary Figure S1B). Therefore, we focused on TRB alone in all further analyses.

To validate these associations in a functional manner, and also to explore the hypothesis that these findings would also be relevant to subclinical hypersensitivity (i.e., silent inactivation), we examined the relationship between TCR diversity at diagnosis and L-asparaginase activity (where inactivation is a functional marker of both overt and subclinical hypersensitivity) in a separate cohort of 113 patients from the Ma-Spore 2020 trial. Here, we found that patients with the occurrence of L-asp inactivation later during treatment had a higher TRB diversity at diagnosis compared to patients with normal L-asp activity throughout treatment (*p* = 0.026) (Figure 1E).

### 3.3. Higher TCR-Beta Diversity Is Characterized by Infrequent, Dynamically Changing, and Less Shared Clonotypes

**Figure 2:**
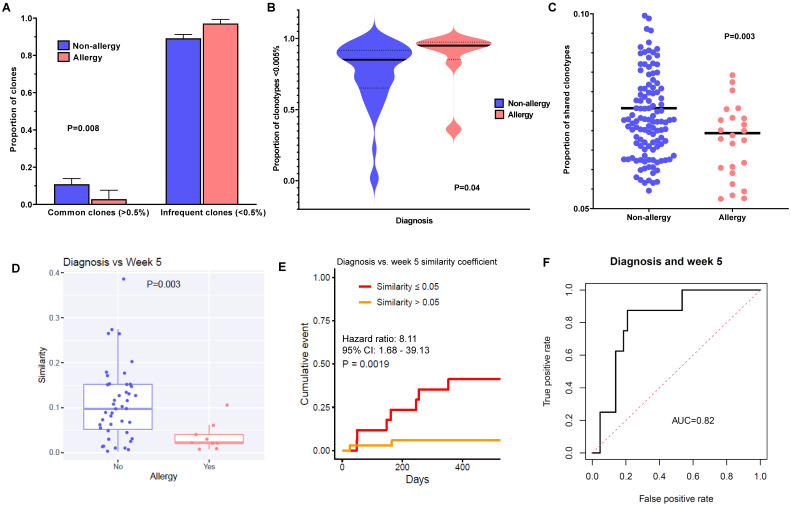
TCR-B diversity in allergic patients is characterized by a higher frequency of infrequently occurring clonotypes, which are less shared amongst patients and display more longitudinal variability: (**A**) Proportion of common (≥0.5%) and infrequent (<0.5%) clones in non-allergy vs. allergy groups. The proportions of common (≥0.5%) and infrequent clones (<0.5%) are shown in the bar chart. Non-allergy is shown in blue, and allergy is shown in red. Allergic patients have a significantly higher proportion of infrequent clones and a lower proportion of common clones. *p*-Values determined by the chi-squared test. (**B**) Proportion of rare clones (<0.005%) in non-allergy vs. allergy groups at diagnosis. The proportions of rare clones are plotted in the violin plots for the non-allergy vs. allergy groups. Non-allergy is shown in blue, and allergy is shown in red. The median in each group is noted as a bold horizontal black line. *p*-Values determined by the Mann–Whitney test. (**C**) Proportion of shared clonotypes in non-allergy vs. allergy groups. The definition of shared clonotypes is as described in the Materials and Methods section. The proportion of shared clonotypes is shown in the dot plot for non-allergy (blue) vs. pre-allergy (green). *p*-Values determined by the Mann–Whitney test. (**D**) Longitudinal similarity of clonotypes in non-allergy vs. allergy groups. Similarity of the TCR repertoires of the same patient between diagnosis and week 5 was measured by the Bhattacharyya similarity coefficient, and the definition is as described in the Materials and Methods section. The similarity coefficients of clones for each patient are plotted in the dot plot for non-allergy vs. allergy. Patients with allergy had significantly more variability between timepoints in their TCR repertoires compared to those without allergy. *p*-Values determined by the Mann–Whitney test. (**E**) Cumulative incidence of allergy in patients with similarity coefficients ≤0.05 vs. >0.05. Similarity of TCR repertoires of each patient between diagnosis and week 5 was measured by the Bhattacharyya similarity coefficient, and the definition is as described in the Materials and Methods section. We divided patients into two groups of similarity coefficients: ≤0.05 (i.e., less similar, shown in red) and >0.05 (i.e., more similar, shown in yellow). There was a significantly higher cumulative incidence of allergy when the similarity coefficient was <0.05, i.e., those who had more variability of clonotypes were at higher risk of allergy, and those with a more static repertoire were at less risk. *p*-Values determined by Cox proportional hazard regression. (**F**) The area under the curve (AUC) of the respective receiver operating characteristic (ROC) curves was calculated for the similarity coefficient of diagnosis vs. week 5.

We sought to characterize the differences in T-cell clonotypes between allergic and non-allergic patients. Since diversity in general could be driven by either increased clonotype richness or higher clonotype evenness, we first evaluated which aspects of TCR diversity were driving this association. We divided the clones into two major frequency groups: common (occurring at a frequency of ≥ 0.5%) and infrequent (< 0.5%, excluding singletons). Here, we found that allergic patients had a higher proportion of infrequent clones (97% vs. 89%, *p* = 0.008; Figure 2A). Further examining within the subset of rare clones (occurring at < 0.005%), we found that allergic patients also had a higher proportion of these clones compared to non-allergic patients (*p* = 0.004; Figure 2B), and this difference was most prominent at diagnosis (85% vs. 95%) compared to week 5 or week 12 (Supplementary Figure S2A). We also examined the proportion of shared clonotypes within this entire patient cohort, where allergic patients had a significantly less sharing of clonotypes between the whole cohort compared to non-allergic patients (7.7% vs. 6.9%, *p* = 0.003; Figure 2C).

Next, we hypothesized that a repertoire that was more dynamic and constantly changing would predispose a patient to increased chances of reactivity, compared to a repertoire that was more static. Therefore, we sought to examine how longitudinal variability of T-cell clones might influence the occurrence of allergy. Using the Bhattacharyya similarity coefficient (where a higher coefficient indicates higher similarity) to compare clonotypes across timepoints, we found that the repertoires of allergic patients were highly dissimilar between diagnosis and week 5 (*p* = 0.003; Figure 2D), whereas patients without allergy tended to retain the same clonotypes. In fact, patients with a similarity coefficient ≤ 0.05 had a significantly higher cumulative incidence of allergy than those with a coefficient > 0.05 (cumulative incidence of allergy 41.2% vs. 5.9%; hazard ratio (HR), 8.11; 95% confidence interval (CI) 1.68–39.13, *p* = 0.001) (Figure 2E), with a relatively strong predictive area under the receiver operating characteristic curve (AUROC) of 0.82 (Figure 2F). We repeated the analyses using the differences for diagnosis vs. week 12 and for week 5 vs. week 12, obtaining similar results, although the AUC was more modest for these subsequent timepoint comparisons (Supplementary Figure S3A,B). Additionally, allergic patients had higher rates of emerging (i.e., “new”) clonotypes at all timepoints as compared to non-allergic patients, who tended to have more persistent (i.e., “old”) clonotypes (Supplementary Figure S4), pointing to increased generation of new clonotypes. Taken together, these findings suggest that a diverse and more dynamically changing clonotypic set in allergic patients would set the milieu for higher risk of subsequently developing L-asp hypersensitivity.

We also assessed the nature of public clonotypes in patients with hypersensitivity, to determine whether there were features of common responses across patients. There were no public clonotypes found against L-asparaginase to suggest a public response (unlike other pathologies, such as viral infections). In fact, we conversely found that allergic patients had fewer public clonotypes across all timepoints compared to non-allergic patients on average (0.43% vs. 0.37% at diagnosis, 0.35% vs. 0.30% at week 5, 0.41% vs. 0.38% at week 12, *p* = 0.016 for overall difference) (Supplementary Figure S5), despite having a higher and more diverse range of clonotypes, i.e., they had more private repertoires. This suggests decreased exposure to common antigens (e.g., viral, bacterial, or fungal) in this cohort of patients, which may predispose them to a higher risk of allergy, consistent with the “hygiene hypothesis” [36], which plausibly plays a role in the development of drug hypersensitivity.

### 3.4. Occurrence of L-Asparaginase Allergy Is Associated with Convergent TCR Response to Common Antigens and Shaping of TCR Gene Usage

**Figure 3:**
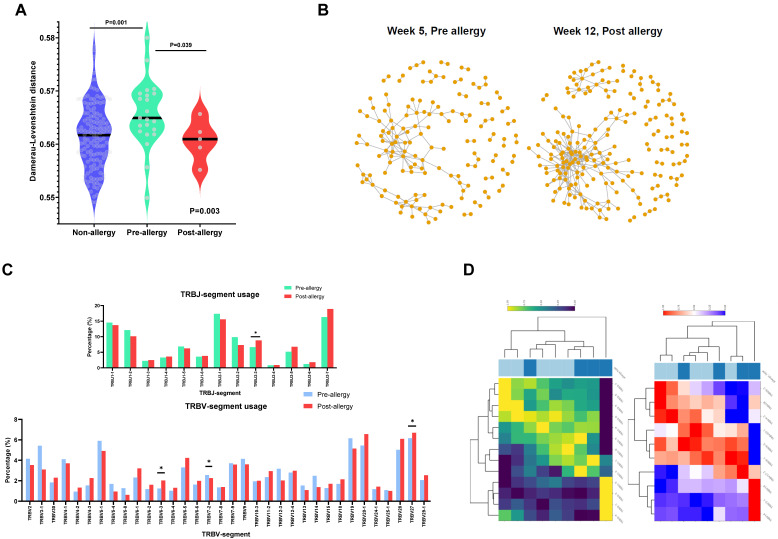
Antigenic shaping of TCR repertoires after the occurrence of asparaginase allergy: (**A**) Interrelation between clones in non-allergy, pre-allergy, and post-allergy groups. The interrelation of clones to one another was determined using the normalized Damerau–Levenshtein distance, which measures the minimum number of substitutions, insertions, and/or deletions necessary to transform one sequence into another. Non-allergy is noted in blue, pre-allergy is noted in green, and post-allergy is noted in red. Clones were less interrelated in pre-allergic patients and became significantly more closely related after the occurrence of allergy, suggesting a convergent response. Overall *p*-values determined by the Kruskal–Wallis test, and *p*-values comparing specific pairs determined by the Mann–Whitney test. (**B**) Clonotypic relationships in a patient before and after grade 4 allergy. In a graphical representation of random sampling of 500 clones, each dot represents a clonotype. Clones that are similar/related are denoted by a gray line linking the two clones. Post-allergy, there is a striking convergence of clonotypes towards one another, indicating a convergent response towards a similar epitope. (**C**) Proportions of TRB-V and TRB-J segment use in pre- and post-allergy. The proportion of each segment’s usage is shown in the bar chart comparing the 4 patients, with sampling performed pre- and post-allergy. Asterisks indicate significant (*p* < 0.05) differential usage according to the Mann–Whitney test. TRB-V: pre-allergy is noted in blue, and post-allergy is noted in red. TRB-J: pre-allergy is noted in green, and post-allergy is noted in red. (**D**) Differentially expressed VJ segment usage. Hierarchical clustering was performed for the top 10 differentially utilized V and J gene segments. The left graph indicates TRB-J (high usage indicated in yellow and low usage indicated in dark blue), while the right graph indicates TRB-V (high usage indicated in red and low usage indicated in blue). The top horizontal panel indicates clustering of pre-allergy (dark blue) and post-allergy (light blue) samples.

We next sought to characterize the nature of the TCR repertoire in patients (N = 4) after the allergy had already occurred. To first assess whether TCR sequences in allergic patients may recognize a common epitope, we evaluated the normalized Damerau–Levenshtein distance between TCR clonotypes (the number of changes of amino acids needed to become the same sequence, normalized by amino acid length, i.e., the closer the distance, the more similar the sequence) and found that post-allergy patients had significantly more closely related clonotypes compared to pre-allergic patients (*p* = 0.039; Figure 3A), indicating a convergence towards a common antigen. In fact, this was most prominent in the patient with grade 4 hypersensitivity (i.e., anaphylaxis), suggesting a correlation with the degree of phenotypic severity (Figure 3B). Comparing the usage of VJ genes between these pre- and post-allergic groups (Figure 3C), we noted a significant decrease in V7-2 (*p* = 0.039) and increases in V6-3 (*p* = 0.034), V27 (*p* = 0.010), and J2-3 (*p* = 0.037). When expanding our analysis to compare all allergic samples vs. non-allergic samples in our cohort, we found additional increased usage of V6-4 (*p* < 0.001) and V15 (*p* < 0.001), and decreased usage of J1-2 (*p* = 0.028), J1-5 (*p* = 0.043), and J2-2 (*p* = 0.004) (Supplementary Figure S6A). Hierarchical clustering of the top 10 differential V and J genes showed segregation of phenotypes, indicating preferential VJ gene usage post-allergy (Figure 3D). Examining TCR features across pre-allergic, post-allergic, and non-allergic patients from the entre cohort, we found that the top 20 clonotypes were highly diverse in both complementarity-determining region 3 (CDR3) sequences and their proportions (Supplementary Figure S6B), with only one common sequence noted from the same patient. Taken together, these findings point to a convergent TCR response to a common antigen, as well as to antigen-driven shaping of the TCR repertoire after the occurrence of allergy.

## 4. Discussion

Although the presence of antibodies has been shown to predict the occurrence of allergy in both L-asp and PEGasp, a significant proportion of allergies (20%) still occur in those without these antibodies [4]. Therefore, more accurate biomarkers to predict these toxicities are still needed. This is especially important in LMICs, where cost-prohibitive alternatives such as Erwinase are difficult to obtain. Here, the presence of increased TRB diversity throughout all earlier timepoints in allergic patients suggests that their preponderance for allergy is contributed to by their pre-existing milieu of immunologically reactive T cells, which establishes the conditions for allergy to occur subsequently. This diversity is characterized by dissimilar and infrequent clonotypes, which we hypothesize increases the overall chance of having a clonotypic sequence that would match to the asparaginase antigen and, thus, could mediate allergy. Therefore, TCR repertoire diversity could potentially be explored as a tool to better predict the occurrence of allergy. One notable index was the similarity coefficient, where the comparison between diagnostic and week 5 samples significantly stratified the cumulative risk of allergy (41% vs. 6%, respectively, with a hazard ratio of 8), with a relatively strong predictive AUC of 0.82. Furthermore, the predictive role of the TCR repertoire in allergy may even be potentially augmented using a combination of T-cell immunity, antibody levels, and L-asp activity. Because current studies mainly address overt clinical hypersensitivity (i.e., grade 2 allergy and above), this would be useful in maximizing therapy in the subsets of patients where L-asp treatment is also suboptimal due to silent inactivation from subclinical hypersensitivity. Silent inactivation represents a significant proportion of patients [2,5] and would go undetected if there is no protocol in place for routine screening of L-asp activity. Our results here point to the influence of TCR diversity on both overt and silent hypersensitivity, and this should be addressed prospectively in a larger cohort of patients, potentially even integrating paired TCR repertoire and L-asparaginase activity assessments. That said, some patients with high TCR diversity did not have any clinical hypersensitivity at all, suggesting that there is interplay with other immune cell populations, e.g., T-regulatory cells, which may mediate or suppress the occurrence of allergy. Interestingly, TCR gamma was not significantly associated with asparaginase allergy at any timepoint, suggesting that the mediation of this phenotype is entirely from TCR alpha–beta chains, and also that gamma–delta T cells—which only comprise a small proportion of total T cells—do not play a significant role in the occurrence of allergy.

The role of immunodominance—i.e., single, dominant T-cell populations responding to a single antigen—has been established in immune responses to viruses and malignancies [37,38]. However, this role is less established in drug hypersensitivity, and it may even differ according to the severity of the reaction, as well as to the drug itself [22,39]. For example, the occurrence of Stevens–Johnson syndrome in carbamazepine (CBZ) has been found to be associated with single and dominant TCR clonotypes directed against CBZ [26,40], but this was not the case for allopurinol-induced hypersensitivity [41]. Although we found here that the TCR response in L-asp allergy had a convergent response to a common antigen, we did not find any specific clonal TCR or motif that was associated with a specific neo-epitope. This might plausibly be attributed to a few reasons. Firstly, since most of our patients only had a mild phenotype, it is possible that large clonal TCR responses that drive more severe phenotypes were not detected in this small group of patients. Secondly, as the majority of TCR sequencing was not performed immediately after the occurrence of an allergy, but rather at fixed serial timepoints, the presence of small-to-moderate clonal reactions might not have been captured—especially after accounting for the lymphodepleting effects of any chemotherapy administered before the sampling.

Strategies to mediate L-asp hypersensitivity include targeting pathways involved in T-cell immunity. For example, genetic or pharmacological inhibition of NFATC2—which reduces T-helper responses—has been shown to provide protection from hypersensitivity reactions to L-asp in mice [12]. However, attenuation of T cells and their diversity needs to be performed in a cautious manner. Decreased TCR diversity may potentially be associated with increased occurrence of viral infections. Furthermore, there is growing evidence to show that the TCR repertoire is correlated with outcomes in several malignancies [42,43]. Therefore, attempting to attenuate the occurrence of hypersensitivity through reducing T-cell immunity might theoretically be associated with the risk of severe infections and/or decreased cure rates. Alternative methods to reduce allergy include modifying the antigenicity of the L-asparaginase protein, or even deriving L-asp through other bacteria [44], although the cost and availability in LMICs where the impact is greatest need to be considered accordingly. The decreased proportion of public clonotypes in allergic patients is consistent with the hygiene hypothesis that decreased antigenic exposure (usually infections) is correlated with a higher incidence of allergic diseases [36]. The mechanisms underpinning this are complex and involve multiple immune factors, including T cells, and are postulated to be due to changes in the gut microbiota that subsequently modulate individual propensity for allergic conditions [45].

One limitation of our study is its relatively small sample size, especially with respect to patients with severe phenotypes. Therefore, future studies with rationally timed longitudinal samples from more patients may more clearly define antigen-driven T-cell responses—particularly those with asparaginase-associated anaphylactic shock, where mediation strategies are more urgently required. Secondly, there is a need for comprehensive human leukocyte antigen (HLA) profiling to be able to clearly define the exact relationships of specific TCR sequences with neoantigens. Our patient cohort was almost entirely Asian, with a comparatively lower rate of allergy than other studies [4] on children of other ancestral descent, e.g., European or African ancestry. Since T-cell immunity is highly entwined with the human leukocyte antigen (HLA) system, which is ancestry-dependent, specific TCRs mediating allergy in one racial/ethnic group may not be broadly generalizable to other races; hence, further studies with comprehensive characterization of HLA in children of diverse populations are warranted.

## 5. Conclusions

In summary, we assessed the role of the TCR repertoire in the occurrence of allergy, characterized its dynamics in patients after asparaginase allergy, and also found factors supporting the hygiene hypothesis in allergic predisposition. Our study further underscores the role of T-cell immunity in this drug allergy and lays the groundwork for understanding the pathophysiology of how T cells mediate allergic responses to asparaginase in children with ALL. Further studies integrating the interactions of both T- and B-cell immune responses, along with functional assessment of asparaginase protein activity, will help pave the way for rationally designed therapies to improve outcomes in children with ALL.

## Figures and Tables

**Table 1:** Clinical profiles of patients with asparaginase-related hypersensitivity.

Patient	Age	Gender	All Subtype	CNS Status	WBC at Diagnosis (× 10^9^/L)	Day of Therapy of Hypersensitivity Occurrence (Phase of Therapy)	Grade of Asparaginase Hypersensitivity	Date of Rechallenge	Grade of Hypersensitivity to Rechallenge	Subsequent Treatment
1	8	F	B Other	CNS I	3.7	161 (Reinduction #1)	2	-	-	Switched to Erwinase
2	5	M	ETV6- RUNX1	CNS I	8.9	48 (Consolidation)	2	3 days after first allergy	2	Switched to Erwinase after 2nd episode
3	2	F	B Other	CNS I	57.0	353 (Reinduction #3)	4	-	-	Switched to Erwinase
4	1	F	KMT2A	CNS I	5.1	391 (Reinduction #3)	2	-	-	Switched to Erwinase
5	6	M	ETV6- RUNX1	CNS I	8.0	165 (Reinduction #1)	2	-	-	Switched to Erwinase
6	3	M	Hyperdiploid	CNS I	8.4	174 (Reinduction #1)	2	-	-	Omitted asparaginase
7	5	F	ETV6- RUNX1	CNS I	1.2	246 (Reinduction #2)	2	-	-	Omitted asparaginase
8 *	1	F	KMT2A	CNS I	120.7	255 (Reinduction #2)	2	2 weeks after first allergy	2	Switched to Erwinase after 3rd episode
9	2	F	Hyperdiploid	CNS I	3.3	49 (Consolidation)	4	-	-	Switched to Erwinase
10	11	M	B Other	CNS II	11.0	25 (Induction)	2	3 weeks after first allergy	4	Switched to Erwinase after 2nd episode
11	1	M	B Other	CNS I	23.0	147 (Reinduction #1)	2	-	-	Omitted asparaginase
12	5	M	Hyperdiploid	CNS I	2.0	49 (Consolidation)	2	-	-	Switched to Erwinase

* Patient 8 developed a 2nd episode of hypersensitivity (grade 3) 2 weeks after rechallenge.

## Data Availability

The data generated in this study are not publicly available due to ongoing clinical trial and data accrual, but they are available upon reasonable request from the corresponding author.

## Supplementary Materials

The following supporting information can be downloaded at: https://www.mdpi.com/article/10.3390/cancers15061829/s1.
