# Supplementary material for: Associations of T-Cell Receptor Repertoire Diversity with L-Asparaginase Allergy in Childhood Acute Lymphoblastic Leukemia
Source: Cancers (Basel). 2023 Mar 17;15(6):1829. doi: 10.3390/cancers15061829 (PMC10047007; doi:10.3390/cancers15061829)
Supplement: Supplementary file 1 [file cancers-15-01829-s001.zip › cancers-2229383-supplementary.pdf]

Supplementary Figures and Tables

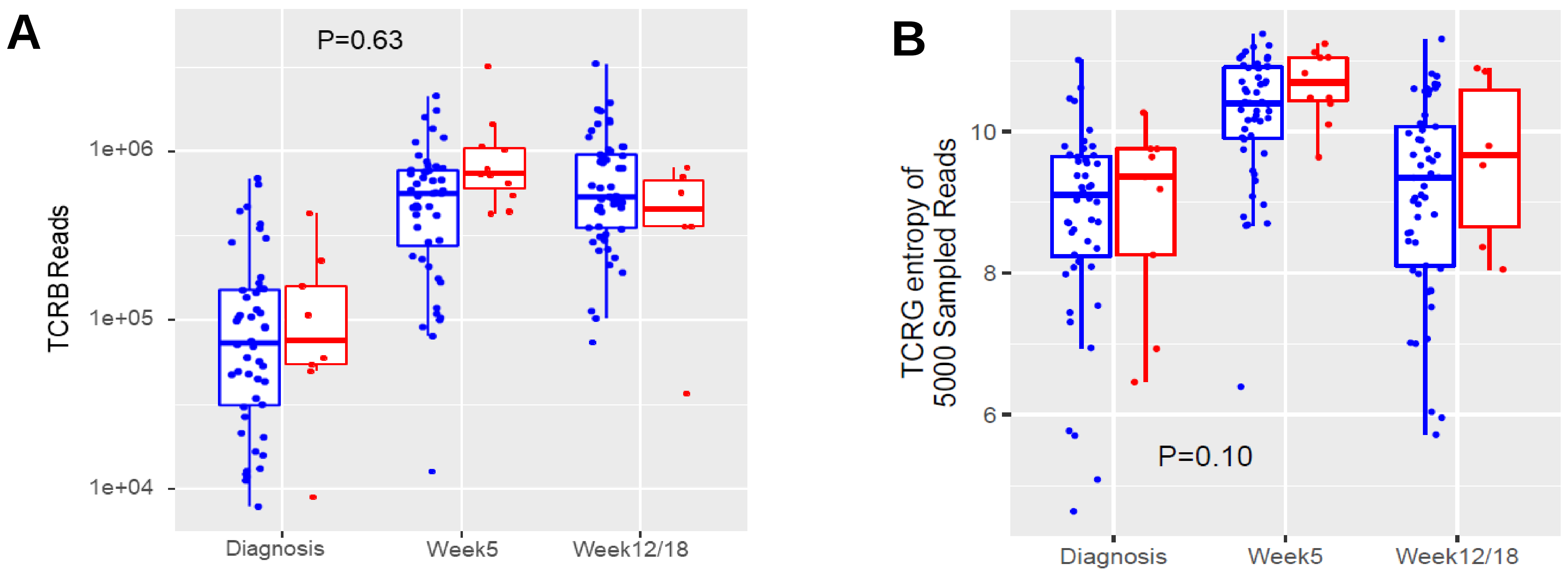

Supp Figure S1. TCR-B and TCR-G diversity in L-asparaginase hypersensitivity

(A) TCR-beta reads in non-allergy vs. allergy across all time-points. TCR-beta reads are plotted for each patient in the scatter plot at each time-point . There are no significant differences in TCR-beta reads between these two groups throughout all time-points of sampling. P-values determined by Mann-Whitney test.

(B) Shannon's entropy of TCR-gamma in non-allergy vs allergy across all time-points. Shannon's entropy of the TCR-gamma repertoire is plotted for each patient in the scatter plot at each time-point and compared between pre-allergic and non-allergic patients. There are no differences in TCR-gamma entropy throughout all time-points, indicating an equal TCR-gamma diversity between these two groups. P-values determined by Mann-Whitney test.

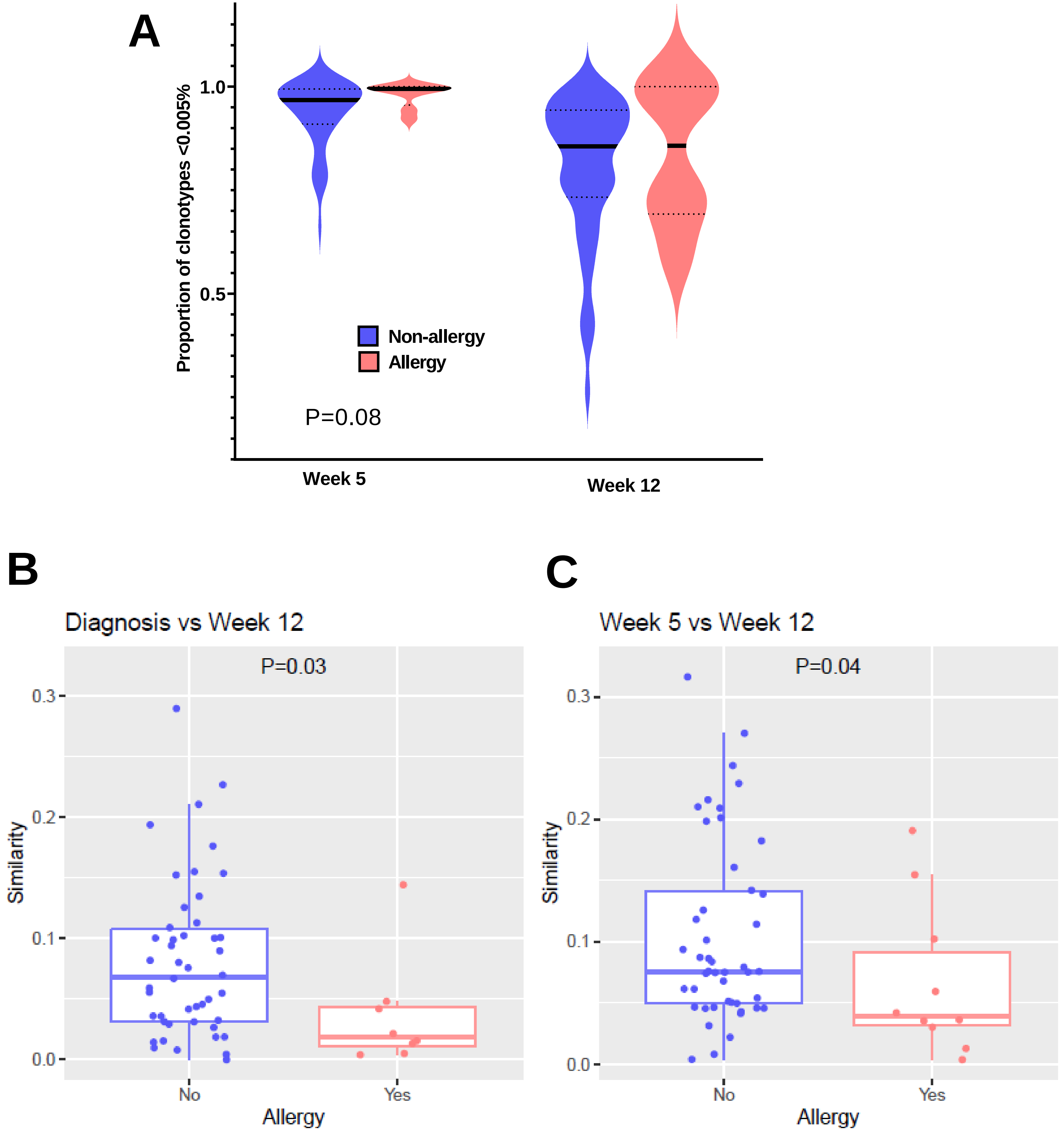

A
Proportion of clonotypes <0.005%
1.0
0.5
Non-allergy
Allergy
P=0.08
Week 5
Week 12
B
Diagnosis vs Week 12
P=0.03
Similarity
0.3
0.2
0.1
0.0
No
Yes
Allergy
C
Week 5 vs Week 12
P=0.04
Similarity
0.3
0.2
0.1
0.0
No
Yes
Allergy

Supp Figure S2. TCR-B diversity is characterized by higher frequency of uncommon clonotypes which are less shared and display more longitudinal variability

(A) Proportion of rare clones (<0.005%) in non-allergy vs. allergy at week 5 and week 12.
The proportion of rare clones are plotted in the violin plots for non-allergy vs. allergy. Non allergy is shown in blue and allergy is shown in red. The median in each group is noted as a bold horizonal black line. P-values determined by Mann-Whitney test.

(B)-(C) Longitudinal similarity of clonotypes in non-allergy vs allergy
Similarity of TCR repertoire between diagnosis vs. week 12 (B) and week 5 vs. week 12 (C) of each patient was measured by the Bhattacharyya similarity coefficient, and the definition is as described in Methods. The similarity coefficient of clones for each patient are plotted in the dot plot for non-allergy vs allergy. Patients with allergy had a significantly more variability between time-points in their TCR-repertoire compared to those without allergy. P-values determined by Mann-Whitney test.

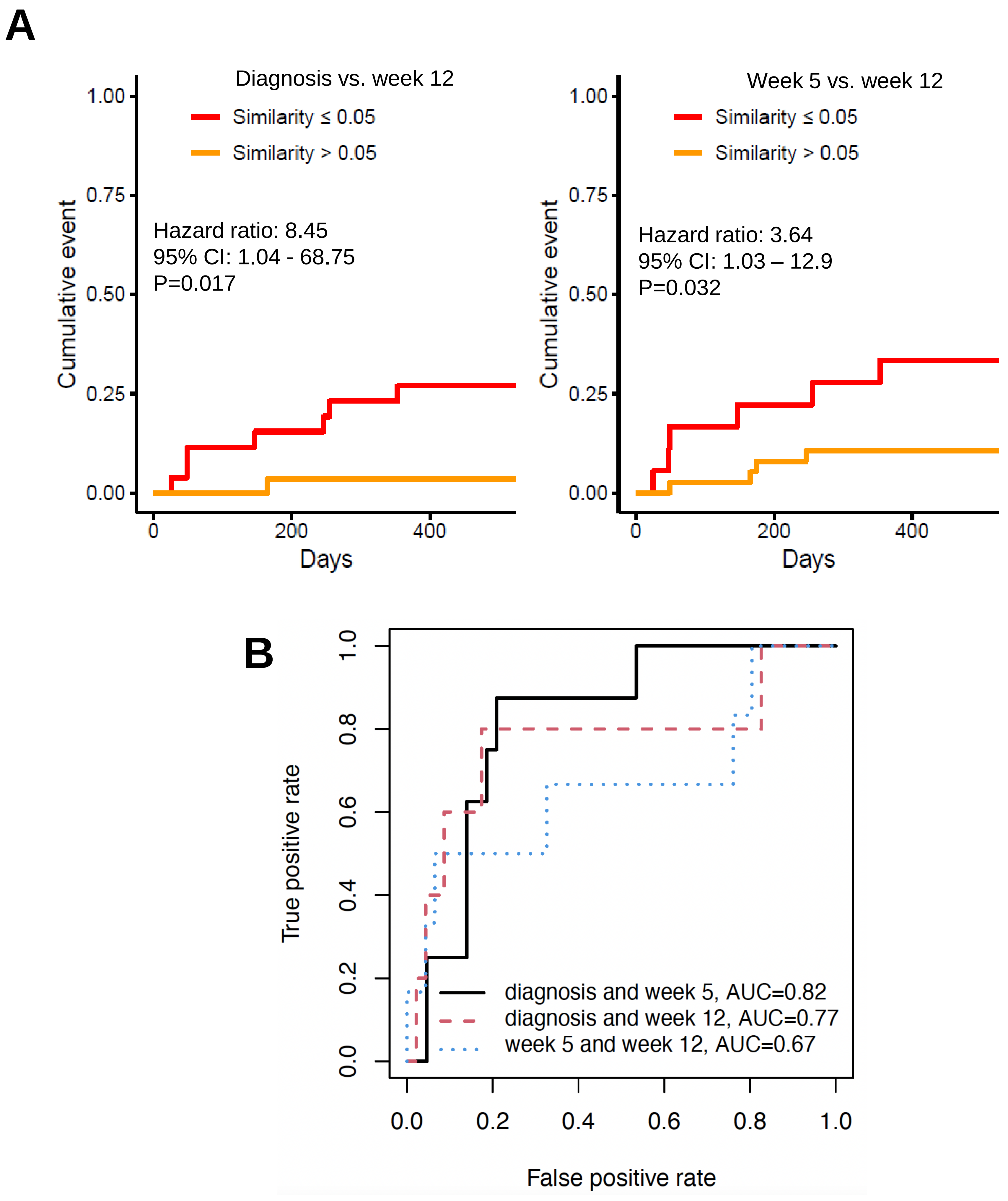
A
Diagnosis vs. week 12
Similarity ≤ 0.05
Similarity > 0.05
Hazard ratio: 8.45
95% CI: 1.04 - 68.75
P=0.017
Cumulative event
1.00
0.75
0.50
0.25
0.00
0
200
400
Days
Week 5 vs. week 12
Similarity ≤ 0.05
Similarity > 0.05
Hazard ratio: 3.64
95% CI: 1.03 – 12.9
P=0.032
Cumulative event
Days
B
True positive rate
False positive rate
0.0
0.2
0.4
0.6
0.8
1.0
diagnosis and week 5, AUC=0.82
diagnosis and week 12, AUC=0.77
week 5 and week 12, AUC=0.67

Supp Figure S3. Cumulative incidence of allergy in patients with similarity coefficient ≤0.05 vs. >0.05

(A) Similarity of TCR repertoire between diagnosis vs. week 12 (left) and week 5 vs. week 12 (right) of each patient was measured by the Bhattacharyya similarity coefficient, and the definition is as described in Methods. We divided patients into two groups of similarity coefficients: ≤0.05 (i.e. less similar, shown in red) and >0.05 (i.e. more similar, shown in yellow). There was a significantly higher cumulative incidence of allergy when the similarity coefficient was <0.05, i.e. those who had more variability of clonotypes were at higher risk of allergy, and those with a more static repertoire were at less risk. P-value determined by Cox-proportional hazard regression.

(B) The area under the curve (AUC) of the respective receiver operator characteristic (ROC) curves are calculated for diagnosis vs week 5 (black), diagnosis vs. week 12 (blue), and week 5 vs week 12 (red). The similarity coefficient at diagnosis vs week 5 has the strongest predictive power for occurrence of allergy, and more modest for the later timepoints.

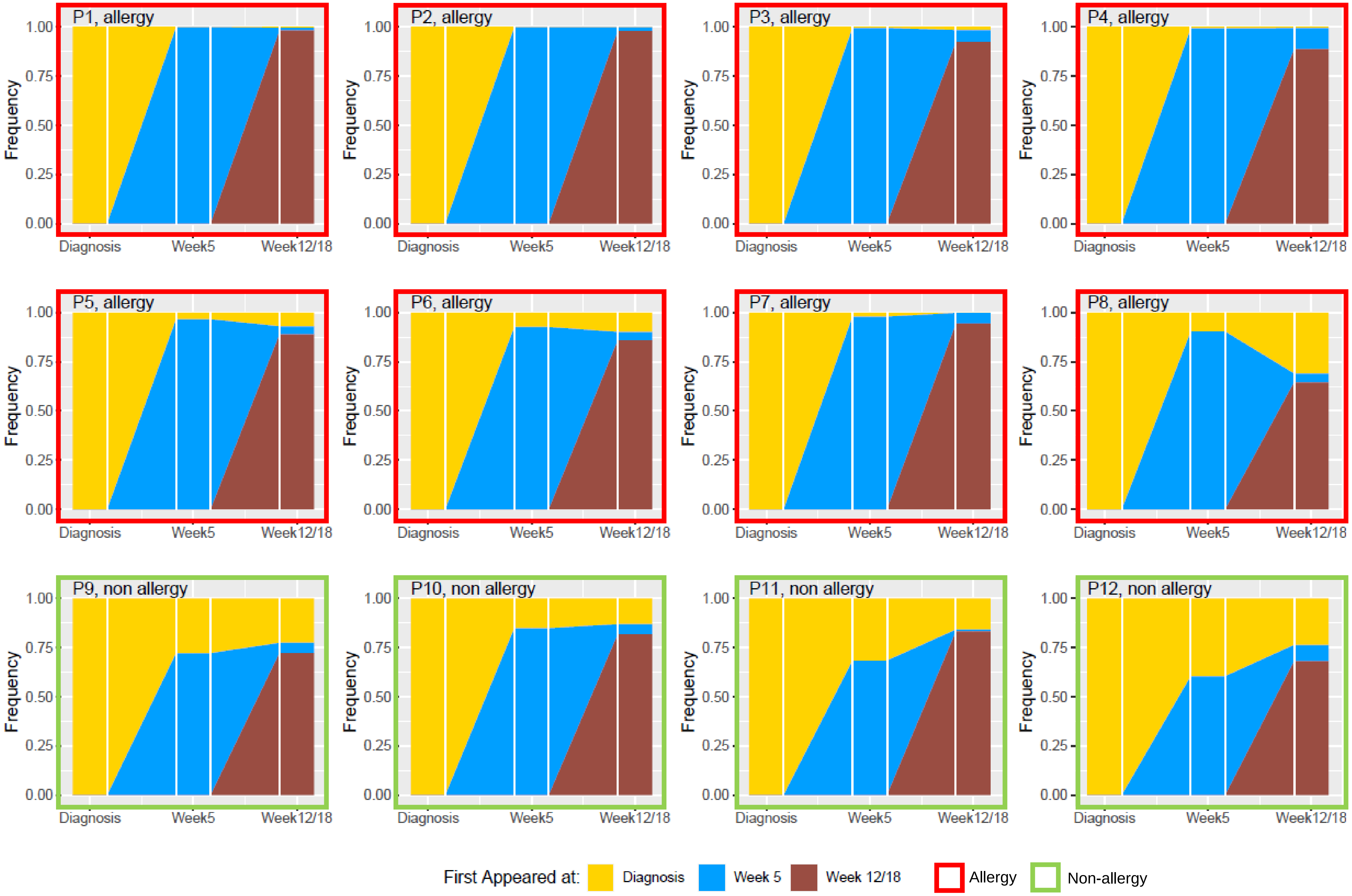

Supp Figure S4. Emergence of new clones at all time-points in patients with allergy vs non-allergy

For each patient, the proportion of clones emerging at each time-point and persisting subsequent are shown. Clones appearing at diagnosis are shown in yellow, at week 5 in blue, and at week 12 in brown. Patients with allergy (P1 to P8, red box) tended to have a higher proportion of emerging “new” clones at both week 5 and week 12, and less persistence of “old” clonotypes from diagnosis, whereas the converse is true for patients without allergy (P9 to P12, green box) who tended to have persistent old clones and less generation of new clones.

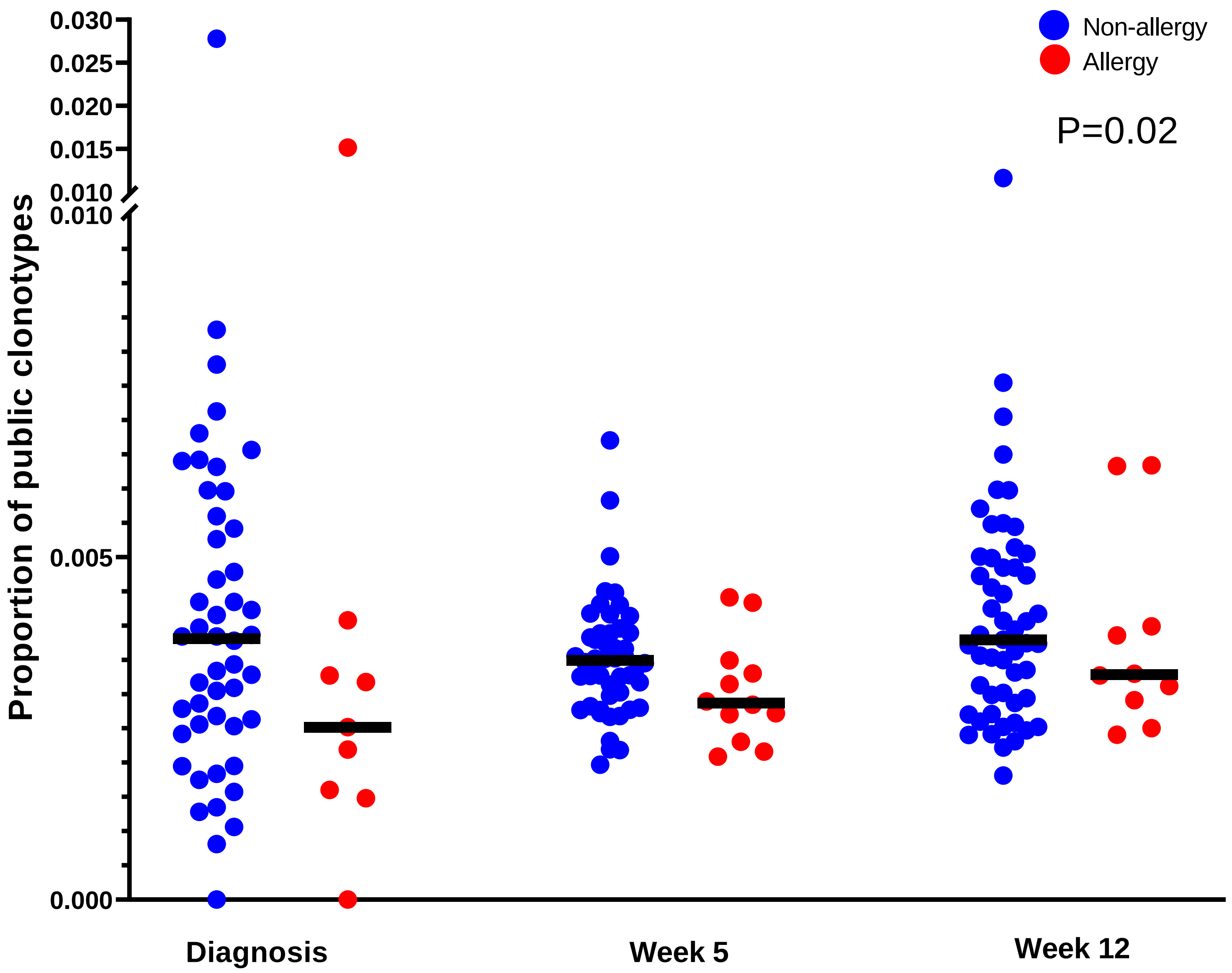

Supp Figure S5. Public clonotypes in allergy vs. non-allergy

The proportion of public clonotypes are plotted at each time-point for each patient in the scatter plot. Allergy is noted in red and non-allergy is noted in blue. Public clonotypes are sequences that are defined in Methods. Here, we find that patients who had allergy have less public clonotypes at all timepoints, even though they have a more diverse set of clonotypes. This is supportive of the “hygiene hypothesis” - decreased common antigenic exposure which may predispose them to a higher risk of allergy. P-value determined by Mann-Whitney test.

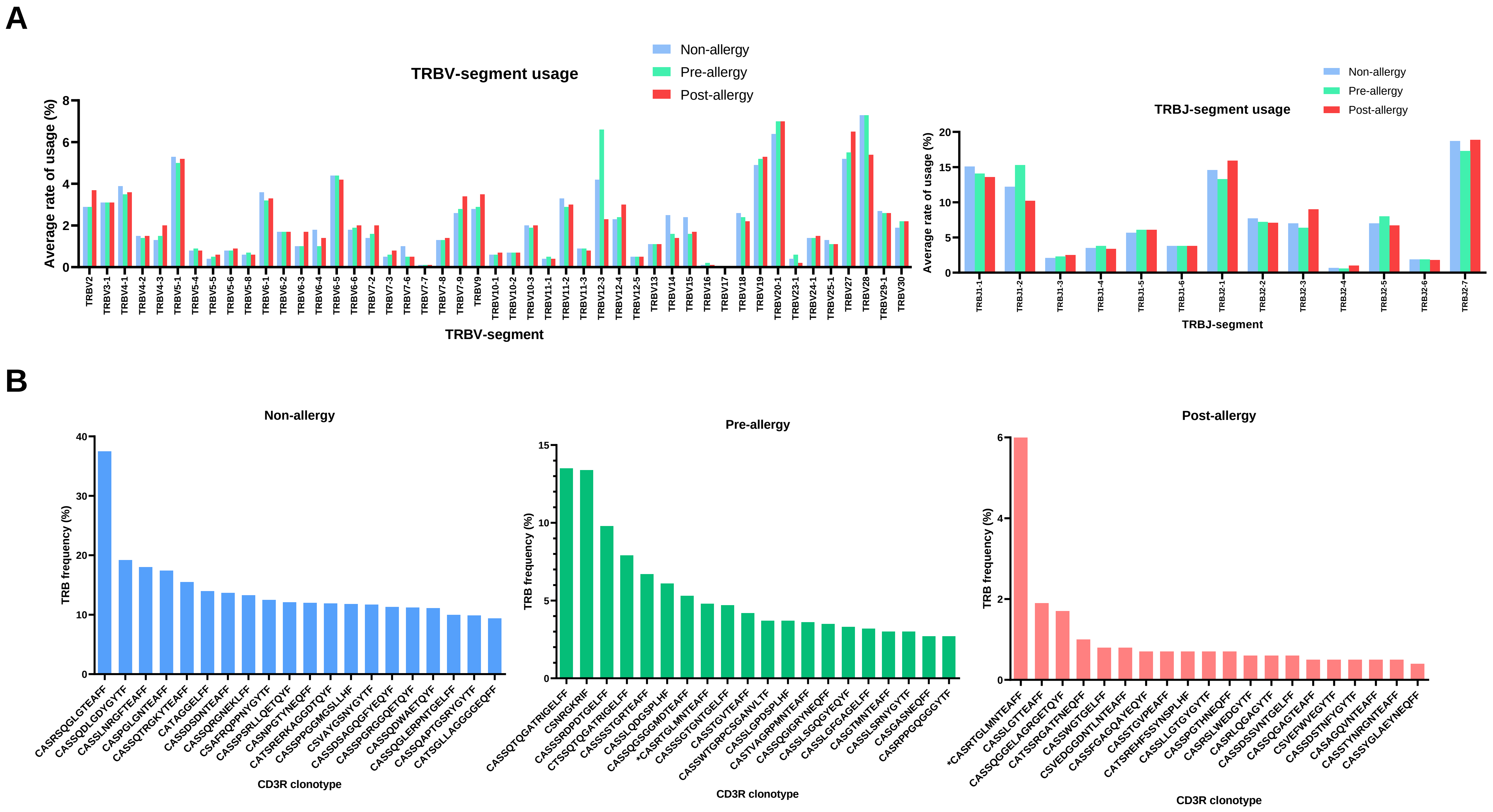

Supp Figure S6. VJ and CDR3 segment use across non-allergy, pre-allergy and post-allergy in entire cohort

(A) Proportion of TRB-V and TRB-J segment across the entire cohort

The proportion of each segment usage is shown in the bar chart comparing non-, pre- and post-allergy. Non-allergy is noted in blue, pre-allergy is noted in green and post-allergy noted in red.

(B) Top 20 CD3R sequences in non-allergy, pre-allergy and post-allergy

The proportion of usage of the top twenty CDR3 sequences for each group are shown in the bar charts. Non-allergy is noted in blue, pre-allergy is noted in green, and post-allergy noted in red. Shared CDR3 sequences are marked by an asterix

Supp Table S1. Comparison of characteristics between allergic and non-allergic patients

		**All patients (N=67)**	**%**	**No allergy (N=55)**	%	**Allergy (N=12)**	%	P-value
Sex	F	27	40.3	22	40.0%	6	50.0%	0.52
	M	40	59.7	33	60.0%	6	50.0%	
Age group (years)	<1	2	3.0	0	0.0%	2	16.7%	0.001
	1 to <10	40	59.7	31	56.4%	9	75.0%	
	≥10	25	37.3	24	43.6%	1	8.3%	
WBC at diagnosis (x 10^9/L)	<50	45	67.2%	35	63.6%	10	83.3%	0.19
	≥50	22	28.8%	20	36.4%	2	16.7%	
Race	Chinese	27	40.2	24	43.6%	5	41.7%	0.14
	Malay	19	28.3	17	30.9%	1	8.3%	
	Indian/Others	21	31.4	14	25.5%	6	50.0%	
